# Spatial interplay of tissue hypoxia and T-cell regulation in ductal carcinoma in situ

**DOI:** 10.1038/s41523-022-00419-9

**Published:** 2022-09-15

**Authors:** Faranak Sobhani, Sathya Muralidhar, Azam Hamidinekoo, Allison H. Hall, Lorraine M. King, Jeffrey R. Marks, Carlo Maley, Hugo M. Horlings, E. Shelley Hwang, Yinyin Yuan

**Affiliations:** 1 Centre for Evolution and Cancer, Institute of Cancer Research, London, UK; 2 Division of Molecular Pathology, Institute of Cancer Research, London, UK; 3 Department of Pathology, Duke University School of Medicine, Durham, NC USA; 4 Department of Surgery, Duke University School of Medicine, Durham, NC USA; 5 Arizona Cancer Evolution Center, Biodesign Institute and School of Life Sciences, Arizona State University, Tempe, AZ USA; 6 Department of Pathology, The Netherlands Cancer Institute, Plesmanlaan, 121 1066CX Amsterdam, The Netherlands

**Keywords:** Breast cancer, Cancer imaging

## Abstract

Hypoxia promotes aggressive tumor phenotypes and mediates the recruitment of suppressive T cells in invasive breast carcinomas. We investigated the role of hypoxia in relation to T-cell regulation in ductal carcinoma in situ (DCIS). We designed a deep learning system tailored for the tissue architecture complexity of DCIS, and compared pure DCIS cases with the synchronous DCIS and invasive components within invasive ductal carcinoma cases. Single-cell classification was applied in tandem with a new method for DCIS ductal segmentation in dual-stained CA9 and FOXP3, whole-tumor section digital pathology images. Pure DCIS typically has an intermediate level of colocalization of FOXP3+ and CA9+ cells, but in invasive carcinoma cases, the FOXP3+ (T-regulatory) cells may have relocated from the DCIS and into the invasive parts of the tumor, leading to high levels of colocalization in the invasive parts but low levels in the synchronous DCIS component. This may be due to invasive, hypoxic tumors evolving to recruit T-regulatory cells in order to evade immune predation. Our data support the notion that hypoxia promotes immune tolerance through recruitment of T-regulatory cells, and furthermore indicate a spatial pattern of relocalization of T-regulatory cells from DCIS to hypoxic tumor cells. Spatial colocalization of hypoxic and T-regulatory cells may be a key event and useful marker of DCIS progression.

## Introduction

Ductal carcinoma in situ (DCIS) of the breast is the most common mammographically detected breast cancer. This “pre-invasive” lesion may progress to invasive ductal carcinoma, but does so at a relatively low frequency (14–53% over 10–15 years)^1^. Nonetheless, it is commonly treated with extensive surgery, radiation, and hormonal therapy even though most of these lesions would never progress to invasive ductal carcinoma. Thus, there is a pressing clinical need to stratify the risk of DCIS tumors into those in need of intervention and those that can be safely monitored without intervention.

Characterizing the evolvability of DCIS into invasive ductal carcinoma could address this need, by predicting those that have a high likelihood of evolving to malignancy versus those that are likely to remain indolent. A recent study based on evolutionary genomic models has categorized DCIS evolution to invasive ductal carcinoma into four models, highlighting its heterogeneity^2^. Since genomics is not the sole driver of tumor behavior, phenotypic characterization of DCIS and its tumor microenvironment, using markers of hypoxia, immune response, and matrix organization among others, will help unveil the influences of the ecological forces driving the evolution of DCIS.

Recent studies highlighted the importance of tumor-infiltrating lymphocytes in the progression from DCIS to invasive ductal carcinoma^3^ and the risk of local and metastatic recurrences. High-grade DCIS contains a higher percentage of FOXP3+ cells compared to the non-high-grade DCIS^4,5^. Consistent with observation in invasive ductal carcinoma that high numbers of FOXP3+ regulatory T cells (Tregs) relative to CD8+ T cells predicts decreased progression-free survival and overall survival^3,6^, an increase in the numbers of FOXP3+ Tregs or a decrease in CD8/FOXP3 ratio in DCIS are associated with increased recurrence risk^3,6,7^. While these studies underscore the role of FOXP3+ T cells in the evolution of DCIS, the low frequency of these cells in DCIS (<10% of all T cells) and their highly variable topological distribution make quantitative assessment and immune scoring a challenging task^8^.

Furthermore, factors promoting Treg cell recruitment in DCIS remain elusive. Hypoxia, a condition defined as lacking or low in oxygen, has been shown to be increased in IDC (invasive regions adjacent to ducts in IDC/DCIS samples) compared with DCIS^9^. It has also been shown to modulate the differentiation and function of T lymphocytes and mediate recruitment of suppressive and proangiogenic T-cell subsets^10^. In invasive ductal carcinoma, hypoxia measured by CA9 positivity has been shown to promote the recruitment of Tregs defined using FOXP3-positive cells in both basal and non-basal subtypes^11^. In DCIS, CA9 was found to be expressed more frequently in high-grade DCIS associated with central necrosis, compared with low-grade DCIS and normal epithelium^9^. However, very little is known about the interplay between hypoxia and T-cell regulation in DCIS and how this influences the progression from DCIS to invasive ductal carcinoma.

With advancing computing techniques, remarkable progress in machine learning has been made on the objective assessment of cellular context in digitized histological sections. Histological samples can provide the spatial context of diverse cell types coexisting within the microenvironment. Advanced computer-vision techniques have been developed for spatial mapping of cells in histological samples. This has enabled the applications of experimental and analytical tools from ecology to cancer research, generating system-level knowledge of microenvironmental spatial heterogeneity.

To enable spatial mapping of hypoxia and T-cell regulation in DCIS, we designed an end-to-end deep learning framework for histology image analysis. We hypothesize, and provide preliminary data, that Treg recruitment is spatially dependent on the hypoxic state. Our primary aims were: (1) to develop a fully automated and versatile pipeline for digital pathology that can accurately classify cells based on hypoxic status and T-regulatory cell marker in immunohistochemistry (IHC) samples; (2) to tailor a powerful deep learning approach for DCIS, thereby dissecting the complex tissue architecture of DCIS. This enabled us to incorporate information from single-cell protein expression, identified in Aim 1, with global DCIS ductal architecture in whole-section tumor sections; (3) to compare the spatial dependency of T-cell regulation on the hypoxic microenvironment in pure DCIS cases and DCIS components identified concurrently with invasive cells in invasive ductal carcinoma.

## Results

### Mapping DCIS intra-tumor heterogeneity of CA9 and FOXP3 expression

**Fig. 1:**
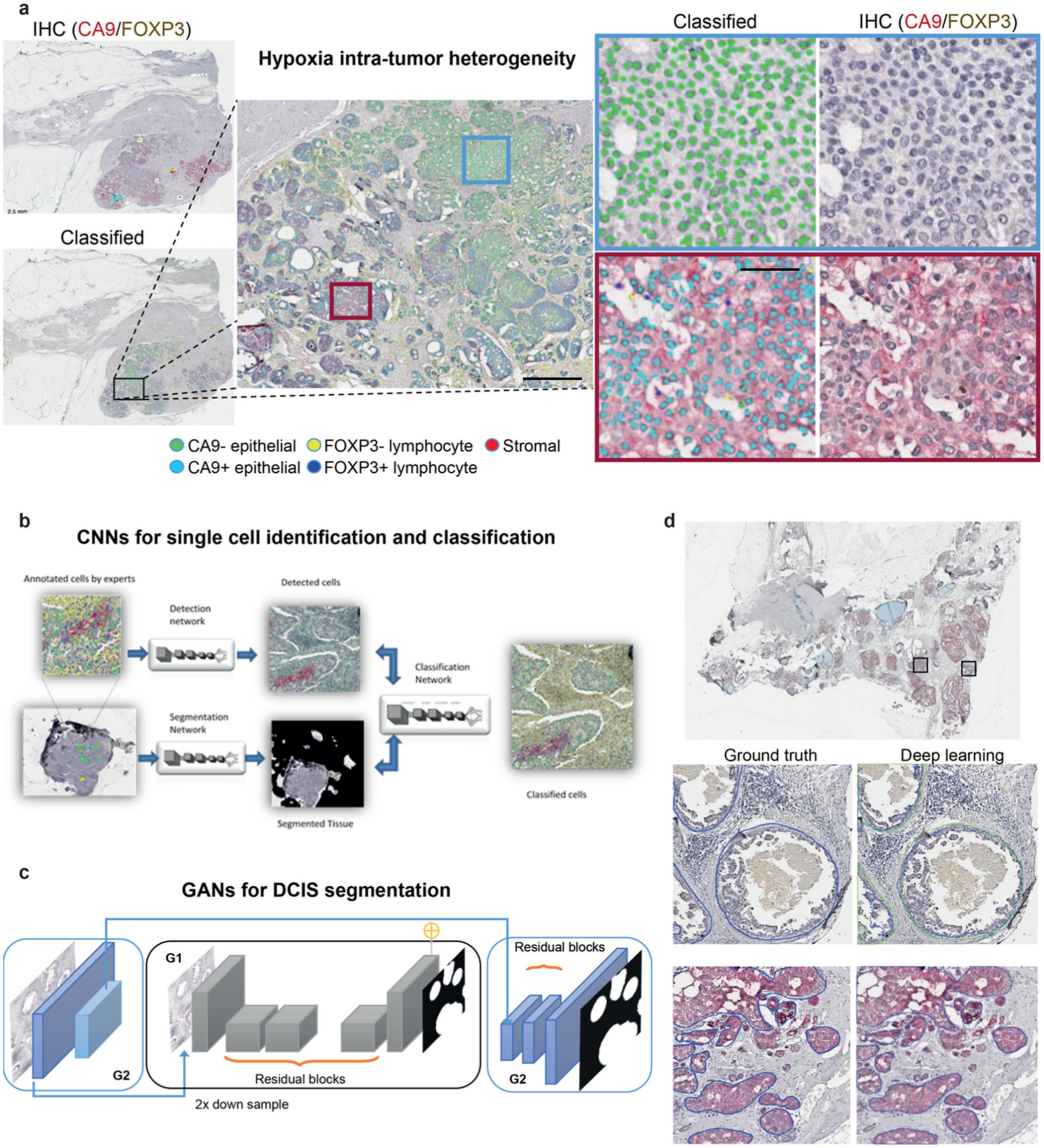
Studying intra-tumor heterogeneity of hypoxia in DCIS using deep learning and digital pathology. **a** An illustrative example of a DCIS tumor with high spatial intra-tumor heterogeneity of hypoxia. Shown are images of IHC dual staining with CA9 and FOXP3, cells were classified into five types based on their expression of CA9 and FOXP3 and morphological features. **b** The deep learning pipeline using convolutional neural networks (CNNs) for single-cell analysis. **c** Generative adversarial networks (GANs) for semantic segmentation of individual DCIS ducts. **d** An example of DICS tumor where individual DCIS ducts have been segmented using GANs. Two high-resolution examples show ground truth obtained from annotations by pathologists and output from GANs. Scale bar represents 100 µm.

To investigate the intra-tumor heterogeneity of tumor hypoxia and T-cell regulation in breast tumors, 99 whole-tumor sections stained with dual marker CA9/FOXP3 from cases clinically classified as pure DCIS (*n* = 30) or invasive carcinomas with synchronous DCIS (IDC/DCIS, *n* = 34) were included in this study (Table 1). Consistent with the previous publications^3,9^ and upon preliminary visual inspection, CA9 expression was rare in normal epithelium and benign lesions, but was present focally in DCIS for both pure DCIS and IDC/DCIS samples. High spatial heterogeneity of CA9 staining in DCIS was evident in some tumors (Fig. 1a).

**Table 1:** Demographics of patients in the dataset comprising pure DCIS and IDC/DCIS samples.

	Pure DCIS (*n* = 30)	IDC/DCIS (*n* = 34)
*Age (median, years)*	61.87	56.29
*Grade*		
1	1	7
2	12	12
3	17	15
*ER status*		
Neg	5	11
Pos	21	23
NP	4	0
*PR status*		
Neg	6	14
Pos	20	20
NP	4	0

**Fig. 2:**
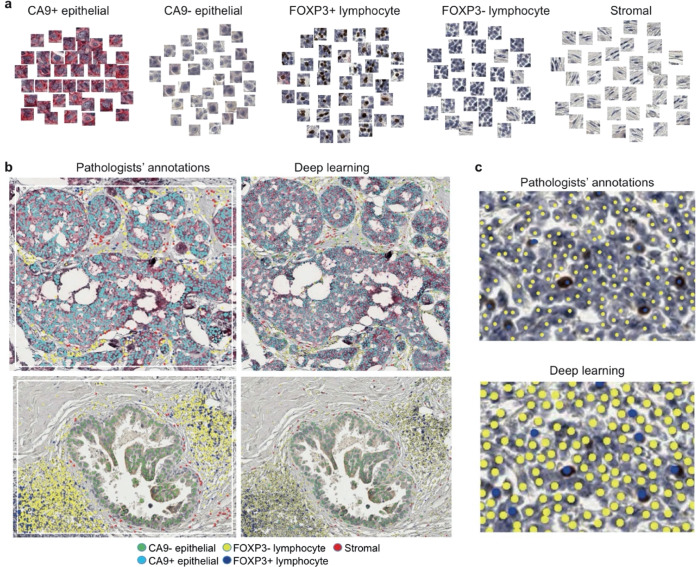
Single-cell classification for CA9/FOXP3-stained immunohistochemistry samples of breast tumors. **a** Examples of five cell classes. **b** Examples of cell annotations by pathologists, collected with white boxes as shown, alongside deep learning output. **c** High-resolution images showing FOXP3– and FOXP3+ lymphocyte examples.

To analyze cell expression in the context of DCIS ductal structure, we designed an end-to-end framework for both single-cell classification and semantic segmentation of DCIS ducts (Fig. 1a–d). For the objective and accurate identification and classification of single cells based on their CA9 and FOXP3 expression, we developed a deep learning approach using convolutional neural networks (CNNs, Fig. 1a, b), using a total of 35,883 single-cell annotations (Methods). Two independent cell identification and three cell classification models were evaluated (Tables 2 and 3, respectively). For single-cell detection, spatially constrained convolutional neural network (SCCNN) outperformed ConCORDe-Net (F1 score = 0.80 for SCCNN and 0.67 for ConCORDe-Net, Table 2). For cell classification, SCCNN achieved the highest test accuracy of 88.6% (accuracy across all cell classes, visual representation in Fig. 2) compared to Inception_v2 and Inception_v3 in a testing set of 10 randomly selected sections and hence was selected as the final model. Confusion matrix of predicted results versus true class (pathologist’s annotations) for five cell classes (CA9+/− epithelial, FOXP3+/− lymphocyte, and stromal cell) are depicted in Table 3. Subsequently, single-cell detection and classification models were applied to all 99 IHC images used in this study, generating 22,121,761 single-cell identities and spatial locations.

**Table 2:** Performance evaluation of deep learning methods for single-cell detection.

Methods	TP	FP	FN	Precision	Recall	F1 score
CONCORD	2582	969	173	0.73	0.62	0.67
SCCNN	3591	1200	564	0.74	0.86	0.80

*TP* number of true positives, *FP* numbers of false positive, *FN* number of false negative, *Precision* percentage of the results that are relevant $\frac{{TP}}{{TP + FP}}$, *Recall* percentage of total relevant results correctly classified by the algorithms $\frac{{TP}}{{TP + FN}}$, *F1 score* harmonic mean of precision and recall $2 \times \frac{{P \times R}}{{P + R}}$.

**Table 3:** Confusion matrix of the prediction results along with the row and column summaries displaying the percentages of correctly and incorrectly classified observations for each true/predicted class.

True class	CA9–	685		28	38	2
	CA9+	2	52	225	6	42
	FOXP3+	12	14	3646		88
	FOXP3–	1	1	14	1195	188
	Stroma	4	2	482	102	2709

Predicted	97.3%	75.4%	83.0%	89.1%	89.4%
	2.7%	24.%	17.0%	10.9%	10.6%
	CA9–	CA9+	FOXP3–	FOXP3+	Stroma

### Deep learning-enabled automated segmentation of DCIS ducts

**Fig. 3:**
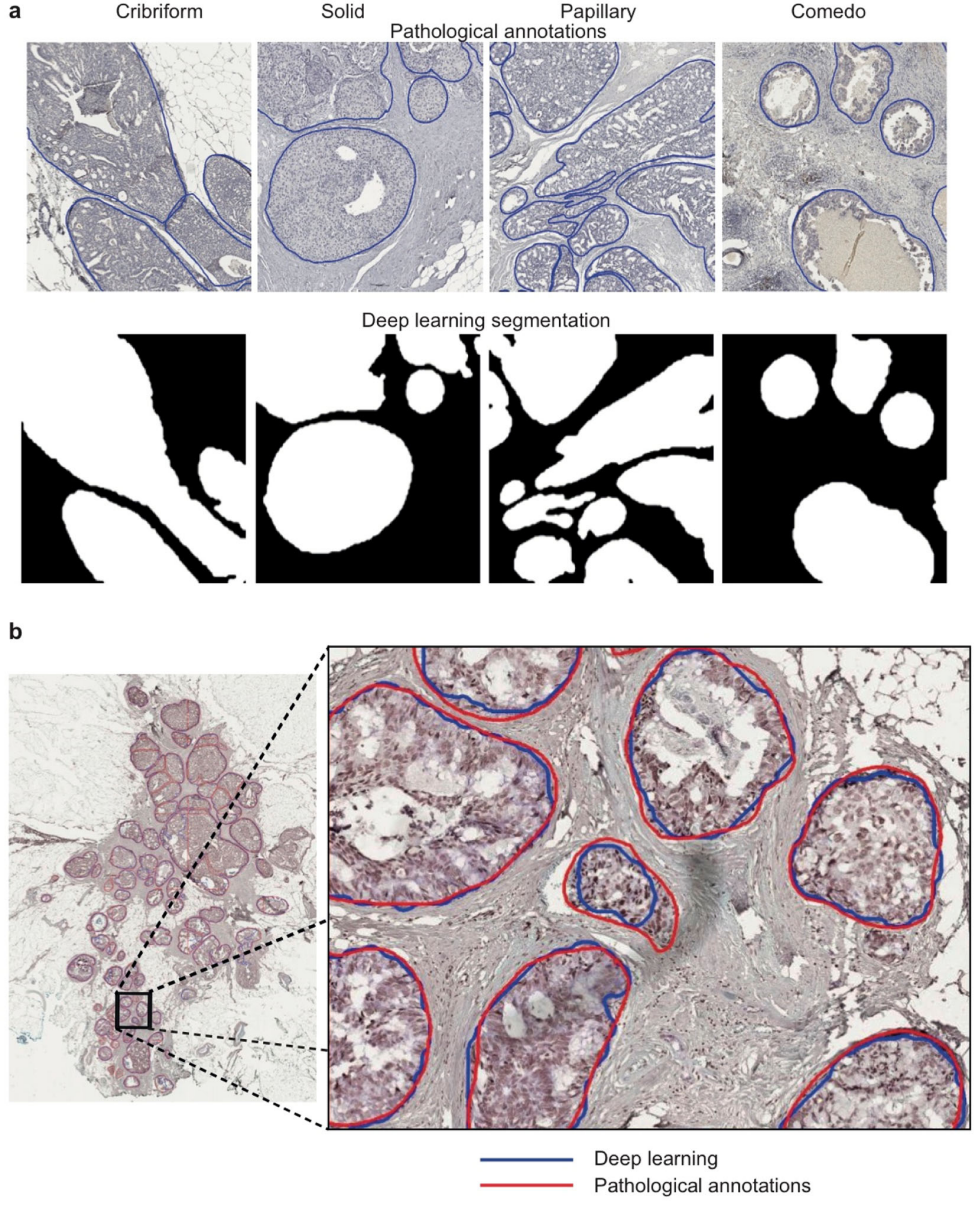
Examples of DCIS duct segmentation in CA9/FOXP3-stained immunohistochemistry samples of breast tumors. **a** Deep learning output according to DCIS histology types. **b** An example of pure DCIS samples, with high-resolution images showing a comparison between pathologists’ DCIS duct annotation (red) and deep learning DCIS duct segmentation (blue).

For the detection and segmentation of DCIS ducts in CA9/FOXP3 IHC images, we developed a new model based on generative adversarial network (GAN), specifically accounting for their complex tissue architecture and highly variable shapes and sizes. (Fig. 1c, d). Given the need to capture large ductal regions as well as the architectural details, we used an extended version of GANs^12^ for analyzing high-resolution histology images and generating semantic label maps corresponding to the target regions (DCIS ducts in our case). This model enabled us to analyze images at high resolutions and predict ducts of variable size and shape (Fig. 3). The network was trained on 18 whole slide images and the performance of the model was tested on annotations from eight unseen slides, using a total of 1500 hand-drawn annotations of individual ducts. To investigate the generalizability of the model, we performed two separate experiments based on different training sets and holdout cross-validation sets (we named them Fold 1 and Fold 2). In each experiment, 18 randomly selected whole slide images were used to make the training set and perform model training. The model performance was then evaluated on the holdout validation set (8 unseen slides) in each experiment. This model achieved the average Dice score of 0.85 and 0.95 for the segmentation performance in the first and the second experiments, respectively. The reported average Dice score is calculated using all the image tiles in the holdout validation set in each experiment (Table 4). The proposed model performs well on the cribriform, solid, and comedo categories with recognizable morphometrics. For example, cribriforms show patterns of gaps between cancer cells. In solid pattern ducts, cancer cells completely fill the affected breast ducts and comedo-type ducts are usually filled by large, markedly cancer cells. The common characteristic of these three DCIS subtypes is that they show distinct boundaries which can be detected better compared to the papillary type ducts. In addition, the limited amount of training data on papillary ducts due to their lower frequency also impacted the performance.

**Table 4:** Evaluation metrics calculated for the DCIS segmentation.

	Dice score	Precision	Recall	Sensitivity	Specificity
Fold 1	0.85	0.89	0.84	0.84	0.98
Fold 2	0.95	0.95	0.94	0.94	0.99

The DCIS segmentation model was applied to IDC/DCIS whole-tumor section IHC images (*n* = 56), identifying and segmenting on average 100 DCIS ducts per section. Notably, the automated DCIS duct segmentation was observed to reliably segment ducts in DCIS histology types such as cribriform, papillary, solid and comedo (Fig. 3). This enabled us to automatically differentiate synchronous DCIS components from IDC components in IDC/DCIS samples, facilitating spatial analysis for individual components in the next step.

### Validating the single-cell classification by comparison with pathological scores of CA9 positivity

As an additional validation, we compared pathologist’s scoring of ductal CA9 positivity (Methods) with abundance of CA9+ cells predicted by deep learning. To this effect, whole-tumor sections with low abundance of deep learning-predicted CA9+ epithelial cells (0–1%, *n* = 80) belonged predominantly (*n* = 74, 92%) to the Pathologist_Score_Negative (samples graded by pathologist as negative for epithelial CA9 expression) with few samples (*n* = 6, 8%) belonging to Pathologist_Score_positive (samples with varying degree of CA9 positivity). The proportion of samples with a higher abundance of deep learning-predicted CA9+ epithelial cells (1–5% and >5%), did not vary between the two pathologist score groups (Table 5). Factors contributing to the discrepancy between deep learning and pathological scoring include cell misclassification by deep learning, the lack of ability of deep learning to adapt to some of the artifacts presented in the slides, such as air bubbles, folds, blurring, etc. In addition, discrepancies when comparing a slide-level assessment with a single-cell-level automated quantification could be due to weak cytoplasmic CA9 expression in a subset of samples. This could explain some of the false-positive deep learning predictions in samples graded negative for CA9 expression by pathologists.

**Table 5:** Comparing the pathological scores of CA9 in DCIS cells with deep learning.

CA9+ abundance (%) (deep learning predicted)	Pathologist_Score_Positive	Pathologist_Score_Negative
0–1% (*n* = 80)	*n* = 6 (8%)	*n* = 74 (92%)
1–5% (*n* = 11)	*n* = 5 (45%)	*n* = 6 (55%)
>5% (*n* = 3)	*n* = 2 (66%)	*n* = 1 (33%)

### Spatial colocalization of CA9 and FOXP3-positive cells in pure DCIS and IDC/DCIS samples

To quantify and compare hypoxia and Treg cell spatial colocalization in the microenvironments of pure DCIS (*n* = 44 IHC images) and IDC/DCIS (*n* = 56) samples, the Morisita–Horn index, an ecological measure of community structure to quantify the extent of spatial colocalization or overlap between two spatial variables^13^ was used. Morisita indices range from 0 to 1, with 0 indicating spatial segregation and 1 indicating maximal colocalization between two spatial variables. For pure DCIS samples, a single value of Morisita colocalization per whole-tumor section was computed. For IDC/DCIS samples, Morisita colocalization for synchronous DCIS (ductal regions in IDC/DCIS samples) and IDC (invasive regions adjacent to ducts in IDCIS samples) were computed per component (Methods).

**Fig. 4:**
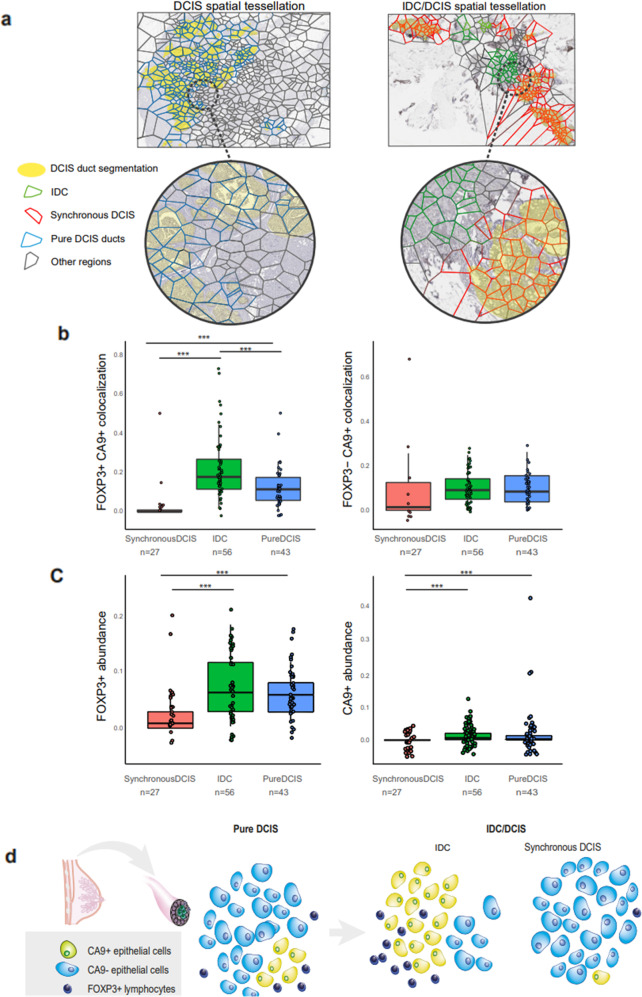
Applying Morisita index to measuring spatial colocalization of cells in DCIS. **a** Voronoi tessellation of tumor region in representative pure DCIS samples (left pane) and IDC/DCIS samples (representative image-right pane), denoting normal DCIS ducts (blue polygons), IDC component (green polygons), and synchronous DCIS (red polygons). Gray polygons (in both DCIS and IDC/DCIS samples) represent other cell types (fibroblasts, normal epithelium, adipose tissue, and artifacts) excluded for analysis. Yellow-shaded regions represent ducts segmented by automated duct segmentation **b** Boxplots comparing colocalization between FOXP3+ and FOXP3– lymphocytes with CA9+ epithelial cells in pure DCIS samples (*n* = 44), IDC (*n* = 56), and synchronous DCIS (*n* = 27) regions. **c** Boxplots comparing abundance of FOXP3+ lymphocytes, CA9+ epithelial cells in pure DCIS samples (*n* = 44), IDC (*n* = 56), and synchronous DCIS (*n* = 27) regions. FOXP3+/− lymphocyte abundance = number of FOXP3+/− lymphocytes/total number of lymphocytes. CA9+/− epithelial cell abundance = number of CA9+/− epithelial cells/total number of epithelial cells. **d** Proposed model for hypoxic epithelial cells promoting Tregs recruitment selected during DCIS progression. ***p < 0.001. *P* values from pairwise Wilcoxon rank-sum test, adjusted using Holm method for multiple testing.

Colocalization between FOXP3+ and FOXP3– lymphocytes with CA9+ and CA9– epithelial cells was compared across the aforementioned sample groups (Table 6 and Fig. 4b). Among the pairwise comparisons (tested using pairwise Wilcoxon rank-sum tests), IDC regions had significantly higher FOXP3+ and CA9+ colocalization, compared to synchronous DCIS regions (*p* = 0.0004) and pure DCIS samples (*p* = 0.0007) (Table 6). Similarly, and consistent with previous reports^5,14^, we report significantly higher FOXP3+ lymphocytes abundance (number of FOXP3+ lymphocytes/total number of lymphocytes) in IDC than in synchronous DCIS regions of the IDC/DCIS samples (*p* = 3.8e–6), indicating a preferential localization of FOXP3+ lymphocytes to the invasive components in IDC/DCIS (Fig. 4c). To ensure that cellular abundance and clinical covariates do not confound colocalization, we report that the difference in FOXP3+ CA9+ colocalization between IDC regions and pure DCIS samples is independent of abundance of FOXP3+ lymphocytes and CA9+ epithelial cells (which does not vary significantly between these groups: *p* = 0.44 and *p* = 0.48 respectively, Fig. 4c), ER status ER status (multivariate *p* value adjusted for ER status = 0.004) and grade (multivariate *p* value adjusted for DCIS grade = 0.01). Consistent with this observation, there was no significant difference between CA9%, FOXP3%, or FOXP3-CA9 colocalization between ER– and ER+ subsets (Supplementary Fig. 1).

**Table 6:** Comparison of colocalization between FOXP3+/− lymphocytes with CA9+/− epithelial cells across IDC, synchronous DCIS, or pure DCIS sample groups.

Colocalization between	IDC vs synchronous DCIS	IDC vs pure DCIS	Synchronous DCIS vs pure DCIS
	Pairwise comparisons using Wilcoxon rank-sum test (adjustment method: Holm)		
FOXP3+ and CA9+	0.0004	0.0007	0.0007
FOXP3– and CA9+	0.15	0.60	0.15
FOXP3+ and CA9–	0.04	0.74	0.05
FOXP3– and CA9–	0.04	0.45	0.04

*P* values are reported from Wilcoxon rank-sum test, adjustment method: Holm.

We explored the differential ductal microenvironments between pure DCIS and synchronous DCIS regions, i.e., ducts adjacent to invasive cancer cells. To this effect, pure DCIS samples had significantly higher FOXP3+ CA9+ colocalization (*p* = 0.0007, Fig. 4a) as well as abundance of CA9+ (*p* = 2.3e–9) and FOXP3+ cells (*p* = 4.4e–6, Fig. 4c), compared to synchronous DCIS regions. The difference in this spatial pattern between pure DCIS and synchronous DCIS remained significant after adjusting for FOXP3+ lymphocyte (adjusted *p* = 0.0005) and CA9+ epithelial cell abundance (adjusted *p* = 0.0012). Within the ER+ subsets of these groups, the differences between groups remained significant (Supplementary Fig. 2). In comparison, there was no significant difference in colocalization of FOXP3– lymphocytes with CA9+ epithelial cells between the three groups (Table 6).

Taken together, we report that the spatial microenvironmental phenotype of IDC regions differs from pure DCIS samples, with increased colocalization of FOXP3+ lymphocytes and CA9+ epithelial cells in IDC regions, independent of cellular abundance and ER status. Notably, our study revealed that the ductal microenvironment of pure DCIS and synchronous DCIS vary, with significant differences in spatial organization of hypoxic CA9+ epithelial cells and FOXP3+ lymphocytes.

## Discussion

This study provides evidence that Treg recruitment is spatially dependent on the hypoxic microenvironment in DCIS. Hypoxia is thought to promote the recruitment of T-regulatory cells for increased immune tolerance and immune evasion^10,11^, but there is a lack of data on this in DCIS. Our analysis integrating deep learning, computational pathology, and spatial statistics on a customized IHC panel revealed a spatial pattern of preferential colocalization between FOXP3+ lymphocytes and CA9+ epithelial cells in DCIS. Compared with pure DCIS samples, the degree of CA9+ epithelial cell and FOXP3+ lymphocyte colocalization was significantly higher in the invasive compartment of invasive breast cancer (IDC), but significantly lower in the synchronous DCIS compartment. These differences were independent of the abundance of these cell types. Therefore, our study reiterates differential microenvironments between pure DCIS and IDC compartments^14–17^. However, we also present evidence that the ductal microenvironment of pure DCIS and synchronous DCIS vary, with significant differences in spatial organization of hypoxic CA9+ epithelial cells and FOXP3+ lymphocytes^14–17^. Based on these data, our proposed model is that hypoxic epithelial cells promoting Tregs recruitment are selected during DCIS progression, resulting in stronger, preferential colocalization of these cells within the invasive compartment (Fig. 4d).

This study adds crucial data to the increasing body of evidence that adaptation to hypoxia as a result of evolutionary selection is key to transition from in situ to invasive cancer^9,18,19^. In addition, it suggests that the inflammatory program that hypoxia promotes through the recruitment of Tregs in DCIS are spatially focal events. We speculate that these focal events may influence the invasive potential of individual DCIS ducts and potentiate the invasion of the basement membrane. We are currently testing this hypothesis comparing cohorts of patients with invasive breast cancer recurrence after a diagnosis of pure DCIS, to patients with pure DCIS who have not had a diagnosis of invasive breast cancer.

Other limitations include the lack of data on other immune cell subsets such as myeloid cells, B lymphocytes, and NK cells, which could add further insights to the immune landscape of DCIS; the limitation on sample size, which prevented further analysis with respect to HR status. Studies on more immune subsets, biological processes, and conditions including hypoxia that may drive the evolution of DCIS to invasive cancers are also ongoing in our laboratories.

Nevertheless, our study defined a new hypoxic and immunosuppressive phenotype, which is the increased spatial colocalization pattern of hypoxic and Tregs identified using machine learning, which differs not only between IDC and synchronous DCIS regions but also pure DCIS samples. With additional validation studies, this new phenotype may be a useful biomarker to predict DCIS progression, and further guide patient selection for new therapeutic approaches to target hypoxia^20^.

Our study was enabled by a new deep learning system design. Deep learning-based methodologies have facilitated a variety of applications in pathology, but the results are often limited to low-resolution images and small images such as tissue microarrays. Precision segmentation of DCIS ducts with highly variable shapes and sizes was not possible in these images. In this work, we generated high-resolution results using a deep learning approach with robust adversarial loss and multi-scale architectures for the generator and the discriminator. This method generates unique results given the same inputs leading to robust and reproducible results. Recently, a U-Net-based deep learning method for the automated detection and simultaneous segmentation of DCIS ducts in H&E samples has been published by our group^21^. Interestingly, we found that this pipeline did not transfer well to the IHC domain, potentially due to the confounding color contrast in staining and less definitive features of DCIS, but in a way resonating similar experience for pathologists. Our proposed pipeline specifically designed for IHC based on generative adversarial models can capture architectural details of DCIS amidst color variations in high resolution. Besides facilitating detailed microenvironmental studies of these ducts, it paves the way for new studies of ductal morphology, adding a new dimension to genotype–phenotype analysis.

In summary, this study highlights the importance of immune spatial heterogeneity in hypoxic tumor microenvironments. It warrants further investigation into detailed molecular activities modulating this phenotype, such as the secretion of chemokine such as CCL28 that induces T-regulatory cell recruitment^22^.

## Materials and methods

### Patient cohort

The dataset consists of patient samples composed of pure DCIS disease (“pure DCIS” henceforth) or IDC/DCIS cases containing synchronous DCIS and invasive components (IDC). A total of 99 whole-tumor sections were obtained from formalin-fixed paraffin-embedded blocks from 64 patients. Patient demographics and baseline characteristics of the dataset are summarized in Table 1. Tissue sections of samples with pure DCIS (*n* = 43: 17 sections with 1 section and 13 sections with 2 sections per patient) and IDC/DCIS samples (*n* = 56: 12 sections with 1 section and 22 sections with 2 sections per patient) were stained and digitized (automated Aperio scanner; resolution = 0.5 µm/pixel; magnification = ×20).

The study was approved by the institutional review board of Duke with a waiver of the requirement to obtain informed consent. ER and PR status were obtained from IHC assay performed at the time of diagnosis (Dako ER pharmDx kit and Dako Link autostainer). Grade of pure DCIS (Table 1, column 2) and IDC/DCIS samples (Table 1, column 3) was based on pathology grading of the whole slide and of the invasive compartment of the IDC/DCIS samples^23^, respectively.

### Immunohistochemistry

All 99 whole-tumor sections used in this study were dual-stained for CA9 and FOXP3. Formalin-fixed paraffin-embedded tissues were dewaxed and 5 µm sections cut. Antigen retrieval was performed by steaming in a 1X Citrate buffer (Sigma C9999). Dual staining was performed using the ImmPRESS Duet Double Staining Polymer kit (HRP Anti-Mouse IgG/AP Anti-Rabbit IgG, Vector labs, MP-7724) as per the manufacturer’s instructions. Sections were stained for cytoplasmic CA9 expression (ImmPACT Vector Red, magenta; primary antibody: rabbit anti-CA9, Novusbio #NB100-417) and nuclear FOXP3 expression (ImmPACT DAB, brown; primary antibody: mouse anti-FOXP3, ABCAM #ab20034), followed by hematoxylin counterstain.

### Pathologist’s score of CA9 positivity in DCIS cells

We used a pathologist-generated score (A.H.H.) of CA9 positivity in DCIS cells as a metric to validate the abundance of CA9+ epithelial cells predicted by our deep learning pipeline. The pathologist’s scoring was performed independently, blinded to the deep learning prediction of CA9+ epithelial cells. The pathologist’s score was based on the intensity of CA9 staining of DCIS cells, with 4 categories ranging from 0 (CA9 Negative, no stain) to 3 (High CA9 staining intensity). The percentage of cells (across a slide) pertaining to each category was scored. For ease of comparison with our deep learning pipeline (which predicts CA9+/− and does not factor intensity), we categorized the pathologist’s score into the following groups:

- Pathologist_Score_Negative: samples in which 100% of DCIS epithelial cells were graded negative for CA9 expression (category 0).
- Pathologist_Score_Positive: samples with varying degrees of CA9 positivity in DCIS epithelial cells (0% in category 0 but >1% in categories 1–3).

### Deep learning pipelines for DCIS IHC histology

The deep learning framework used to analyze pure DCIS and IDC/DCIS samples in this study consists of four parts.

### Tissue segmentation

Fully automated tissue segmentation was performed to remove background and reduce noise and artifacts, allowing for computational efficiency and reduced processing time in subsequent image analysis steps. Tissue segmentation was performed using a pre-trained Micro-Net-512^24,25^. Each whole slide image was reduced to ×1.25 resolution, which was subsequently visualized at multiple resolutions in Micro-Net-512 architecture to capture context information and maintain salient features.

### Single-cell identification

A deep learning model was used to identify all individual cells within a given IHC tissue section. The main objective of this step was to detect all nuclei in a whole slide image by locating nuclei center positions, regardless of their class labels. Briefly, an SCCNN^25,26^ was trained to predict the probability of a pixel being the center of a nucleus. The single-cell detection model was trained in a supervised manner based on pathologist-derived single-cell annotations. To this effect, 26,345 single-cell annotations from 10 whole slide images (double stained for CA9 and FOXP3) were collected from a pathologist (H.M.H). Two network architectures, SCCNN^26^ and ConCORDe-Net^27^, were tested and the architecture that produced single-cell detection with the highest accuracy was adapted.

### Single-cell classification

A deep learning model was used to classify single cells (detected in the previous step) into one of five classes: Stroma, FOXP3+ lymphocyte, FOXP3– lymphocyte, CA9+ epithelial cells, and CA9– epithelial cells. Softmax SCNN^26^ network was used to train the single-cell classification model. Briefly, nuclear morphology features such as shape, size, color, and texture were considered as the main parameters for the model to distinguish between different cell classes. The single-cell classification model was trained in a supervised manner based on pathologist-derived (H.M.H) annotations performed on 12 whole slide images. A total of 35,883 single-cell annotations (2580 stromal cells, 1462 FOXP3+ lymphocytes, 15,413 FOXP– lymphocyte, 4229 CA9+ epithelial cells and 12,199 CA9– epithelial cells) were used for model training. Three network architectures: SCCNN, Inception_v2, and Inception_v3 were tested and the architecture that produced single-cell classification with the highest accuracy was adapted.

Whole slide images were split into tiles of size 2000 × 2000 pixels (with pixel size of 0.5 µm/pixel). Quality control of annotations was performed at the tile level and expert consensus was obtained before using the respective tiles for training. Performance of deep learning models for single-cell detection and classification was evaluated in independent samples (not used for training) by comparison with ground truth, i.e., pathologist’s annotations. In the case of the single-cell classification model, a five-fold cross-validation was performed to account for class imbalance and sampling effects. Annotations from ten whole-section tumor images (26,345 cells) were randomly divided into five equal groups. Class imbalance was taken into account while creating these five groups. For each cross-validation, four groups were chosen for training and one group for testing. From the four groups of training data, 20% (5269 cells) of the annotations was randomly picked for validation purpose. Therefore, we trained and tested each classifier five times separately on each of these groups. Three single-cell classification networks were trained on the created training data and their performances on the testing set were compared. Based on our results, the SCCNN obtained the average accuracy of 89%, the Inception_v3 obtained the average accuracy of 86% and the Inception_v2 obtained the average accuracy of 82%. The SCCNN (see Fig. 2) achieved the highest, 89% accuracy for cell classification in the validation set.

### DCIS duct detection and segmentation

We describe a deep learning model for the simultaneous detection and segmentation of DCIS ducts from IHC images. An improved GANs architecture^12^ was used to train a deep learning model capable of delineating DCIS duct regions from surrounding tissue.

### DCIS duct segmentation using GANs

We propose an improved GAN incorporating a coarse-to-fine generator and multi-scale discriminator architectures suitable for conditional image generation at a much higher resolution. This method is based on conditional GANs that use a robust adversarial learning objective together with new multi-scale generator and discriminator architectures. Using this network, the goal is to translate an input histology image from IHC domain to the binary domain given input-output (histology-mask) image pairs used as training data. This model is composed of two main parts: (i) the generator and (ii) the discriminator. The objective of the generator (G) is to generate semantic label maps (in binary) from histology images (in RGB), while the discriminator (D) learns to distinguish ground truth (GT) images from the created masks (semantic label maps). This framework operates in a supervised setting and the training dataset is prepared as sets of pairs of corresponding images {*x*_*i*,*y*_*i*}, where *x*_*i* is a histology image and *y*_*i* is the corresponding ground truth label map, annotated by expert pathologists. The overall aim of the network is to model the distribution of the semantic label maps via Eq. (1), given the input histology tiles.

1 $${\rm{Min}}_G\left( {{\rm{Max}}_{D1,D2,D3}\mathop {\sum}\limits_{k = 1}^{k = 3} {\left\{ {E_{\left( {x,y} \right)}\left[ {{\rm{log}}\,D_k\left( {x,y} \right)} \right] + E_x\left[ {{{{\mathrm{log}}}}\left( {1 - D_k\left( {x,G(x)} \right.} \right)} \right]} \right\}} } \right)$$

In Eq. (1), *G* denotes the generator that contains two sub-networks: (i) G1 or global generator with the aim of generating the initial prediction (semantic map) and (ii) G2 or local generator with the aim of enhancing the quality of the output from G1 and generating the output image with a higher resolution (i.e., ×2). Primarily, a histology image of resolution [1024 × 1024 × 3] is passed through the sequential components of the global generator to output a binary image of resolution [1024 × 1024]. Subsequently, the output of the convolutional front-end of the local generator plus the last feature map of the global generator are fed to the residual block of the local generator, effectively integrating the global information captured by G1. In Eq. (1), *D* denotes the discriminator part that employs multiple discriminators with identical network structures that operate at different scales of an image pyramid. These discriminators are trained to differentiate the GT and predicted masks at different scales, which encourages coarse to fine learning of the generator. We have used three discriminators (*k* = 3) as suggested by^12^, all of which have identical architectures but operate at different scales (coarse to fine). This specific architecture combination helps the generator to learn a better and consistent global view of the image to generate while improving the details of the generated image.

The model was trained on hand-drawn annotations of more than 3600 individual ducts (H.M.H.) pertaining to 18 whole slide images. The performance of the model was evaluated on 1500 hand-drawn individual ducts annotations of (H.M.H.) pertaining to an independent set of 8 whole slide images, i.e., images not used for model training. Segmentation efficiency was evaluated using the Dice score, which estimates the degree of overlap between the ground truth (human-defined duct boundaries) and model prediction of duct boundaries^28,29^.

### Applying trained deep learning models on whole slide IHC samples

The deep learning framework described above was applied to the pure DCIS and IDC/DCIS tissue sections used in this study. However, DCIS segmentation was not applied to pure DCIS samples, since these samples effectively contain only duct regions. The final outcome of the deep learning pipeline was the spatial quantification of five cell classes (stroma, FOXP3+ lymphocyte, FOXP3– lymphocyte, CA9+ epithelial cells, and CA9– epithelial cells) in pure DCIS samples, synchronous DCIS (ducts within IDC/DCIS samples), and IDC.

### Quantifying cellular abundance and colocalization in pure DCIS and IDC/DCIS samples

The deep learning-predicted cell classes and their location were used to compute the abundance of cell types and the degree of colocalization between pairs of cell types, in pure DCIS and IDC/DCIS samples.

Cellular abundance: FOXP3+ and CA9+ abundance was computed as follows and compared across groups

2 $${\rm{FOXP3}} + {\rm{lymphocyte}}\;{\rm{aboundance}} = \frac{{{\rm{number}}\;{\rm{of}}\;{\rm{FOXP3}} + {\rm{lymphocytes}}}}{{{\rm{total}}\;{\rm{number}}\;{\rm{of}}\;{\rm{lymphocytes}}}}$$

3 $${\rm{CA9}} + {\rm{epithelial}}\;{\rm{aboundance}} = \frac{{{\rm{number}}\;{\rm{of}}\;{\rm{CA9}} + {\rm{lymphocytes}}}}{{{\rm{total}}\;{\rm{number}}\;{\rm{of}}\;{\rm{epithelial}}\;{\rm{cells}}}}$$

Cellular colocalization: the Morisita–Horn Index^30^ was used to quantify colocalization of FOXP3+, FOXP3– lymphocytes with CA9+ and CA9– epithelial cells. Briefly, The Morisita–Horn Index computes the statistical significance in spatial co-occurrence of a pair of cell types within a spatial region defined by Voronoi tessellation. The number of seeds needed to perform Voronoi tessellation (prior to computing colocalization) was set as the cube root of all cells within the region of interest.

For the pure DCIS samples which are composed exclusively of DCIS duct regions, cellular abundance and colocalization were computed across whole slide images. This produced a single abundance and colocalization index (per pair of cell types), per whole-tumor section.

IDC/DCIS samples are composed of DCIS ducts (segmented by deep learning DCIS segmentation) as well as invasive components. Given our interest in analyzing each component individually, we adapted the following approach: the entire tissue area was divided into non-overlapping Voronoi polygons which were classified into three types (Fig. 4a): (1) synchronous DCIS polygons composed of only DCIS ducts, (2) IDC polygons composed predominantly of invasive cancer cells and (3) mixed polygons composed of both ducts and invasive regions, denoting the interface between DCIS and invasive regions. Note: synchronous DCIS were named as such to distinguish them from pure DCIS regions in subsequent comparative analyses. Cellular abundance and colocalization were computed separately for synchronous DCIS and IDC regions, producing two distinct abundance/colocalization indices per whole section of tumor images. The discrepancy in the number of synchronous DCIS regions (*n* = 27, rather than 56 to match the IDC regions) is due to a proportion of IDC/DCIS samples (*n* = 29) that did not contain sufficient synchronous DCIS regions for spatial analysis. These samples were not used for comparisons described above, since they produce a null synchronous DCIS colocalization index (owing to the lack of synchronous DCIS regions).

### Statistical tests

Statistical significance of variable colocalization between pairs of cell types (CA9+/−, FOXP3+/−) across any two groups (IDC vs synchronous DCIS, IDC vs pure DCIS, and synchronous DCIS vs pure DCIS) was performed using Wilcoxon rank-sum test (multiple test adjustment method: Holm). Multivariate regression tests were used as tests of independence to account for covariates such as cellular abundance, ER status, where relevant.

### Reporting summary

Further information on research design is available in the Nature Research Reporting Summary linked to this article.

## Supplementary information

Reporting Summary

Supplementary Information

## Data Availability

All training data, including the fully anonymized raw image tiles and pathological annotations, and binary marks, are available in the corresponding author’s https://github.com/sobhani/DCIS-CA9. Requests for data access for the Duke samples can be submitted to E.S.H. (shelley.hwang@duke.edu) and Y.Y. (yinyin.yuan@icr.ac.uk). Data underlying Fig. 4b, c are in the R package in the same GitHub repository https://github.com/sobhani/DCIS-CA9.

## Code availability

The deep learning pipeline for digital pathology image analysis is available in the GitHub repository https://github.com/sobhani/DCIS-CA9 for reproducibility. All codes used for statistical analyses of image data were developed in R (v.3.5.1) and are available at https://github.com/sobhani/DCIS-CA9.
