# Supplementary material for: Spatial interplay of tissue hypoxia and T-cell regulation in ductal carcinoma in situ
Source: NPJ Breast Cancer. 2022 Sep 15;8:105. doi: 10.1038/s41523-022-00419-9 (PMC9477879; doi:10.1038/s41523-022-00419-9)
Supplement: Supplementary file 1 — Reporting Summary [file 41523_2022_419_MOESM1_ESM.pdf]

nature portfolio

Corresponding author(s): Yinyin yuan, Faranak Sobhani

Last updated by author(s): 18/11/2021

Reporting Summary

Nature Portfolio wishes to improve the reproducibility of the work that we publish. This form provides structure for consistency and transparency in reporting. For further information on Nature Portfolio policies, see our Editorial Policies and the Editorial Policy Checklist.

Statistics

For all statistical analyses, confirm that the following items are present in the figure legend, table legend, main text, or Methods section.

n/a	Confirmed	
☐	☒	The exact sample size (n) for each experimental group/condition, given as a discrete number and unit of measurement
☐	☒	A statement on whether measurements were taken from distinct samples or whether the same sample was measured repeatedly
☐	☒	The statistical test(s) used AND whether they are one- or two-sided<br>*Only common tests should be described solely by name; describe more complex techniques in the Methods section.*
☐	☒	A description of all covariates tested
☐	☒	A description of any assumptions or corrections, such as tests of normality and adjustment for multiple comparisons
☐	☒	A full description of the statistical parameters including central tendency (e.g. means) or other basic estimates (e.g. regression coefficient) AND variation (e.g. standard deviation) or associated estimates of uncertainty (e.g. confidence intervals)
☐	☒	For null hypothesis testing, the test statistic (e.g. F, t, r) with confidence intervals, effect sizes, degrees of freedom and P value noted<br>*Give P values as exact values whenever suitable.*
☒	☐	For Bayesian analysis, information on the choice of priors and Markov chain Monte Carlo settings
☐	☒	For hierarchical and complex designs, identification of the appropriate level for tests and full reporting of outcomes
☐	☒	Estimates of effect sizes (e.g. Cohen's d, Pearson's r), indicating how they were calculated

Our web collection on statistics for biologists contains articles on many of the points above.

Software and code

Policy information about availability of computer code

Data collection	All training data, including the fully anonymised raw image tiles and pathological annotations and binary marks, are available in the GitHub repository https://github.com/sobhani/DCIS-CA9. Requests for data access can be submitted to E. Shelley Hwang (shelley.hwang@duke.edu) and Yinyin Yuan (yinyin.yuan@icr.ac.uk).
Data analysis	The deep-learning pipeline for digital pathology image analysis is available in the GitHub repository https://github.com/sobhani/DCIS-CA9 for reproducibility. All code used for statistical analyses of image data was developed in R (v.3.5.1) and is available at https://github.com/sobhani/DCIS-CA9.

For manuscripts utilizing custom algorithms or software that are central to the research but not yet described in published literature, software must be made available to editors and reviewers. We strongly encourage code deposition in a community repository (e.g. GitHub). See the Nature Portfolio guidelines for submitting code & software for further information.

Data

Policy information about availability of data

All manuscripts must include a data availability statement. This statement should provide the following information, where applicable:

- Accession codes, unique identifiers, or web links for publicly available datasets
- A description of any restrictions on data availability
- For clinical datasets or third party data, please ensure that the statement adheres to our policy

All training data, including the fully anonymised raw image tiles and pathological annotations and binary marks, are available in the GitHub repository https://github.com/sobhani/DCIS-CA9. Requests for data access can be submitted to E. Shelley Hwang (shelley.hwang@duke.edu) and Yinyin Yuan (yinyin.yuan@icr.ac.uk).

Field-specific reporting

Please select the one below that is the best fit for your research. If you are not sure, read the appropriate sections before making your selection.

☒ Life sciences ☐ Behavioural & social sciences ☐ Ecological, evolutionary & environmental sciences

For a reference copy of the document with all sections, see nature.com/documents/nr-reporting-summary-flat.pdf

Life sciences study design

All studies must disclose on these points even when the disclosure is negative.

Sample size	The dataset consists of patient samples composed of pure DCIS disease or IDC/DCIS cases containing synchronous DCIS and invasive components (IDC). 99 whole-tumor sections were obtained from formalin-fixed paraffin-embedded blocks from 64 patients. Tissue sections of samples with pure DCIS (n = 43: 17 sections with 1 section and 13 sections with 2 sections per patient) and IDC/DCIS samples (n = 56: 12 sections with 1 section and 22 sections with 2 sections per patient) were stained and digitized (automated Aperio scanner; resolution=0.5 µm/pixel; magnification=20x).
Data exclusions	n/a
Replication	n/a
Randomization	n/a
Blinding	n/a

Reporting for specific materials, systems and methods

We require information from authors about some types of materials, experimental systems and methods used in many studies. Here, indicate whether each material, system or method listed is relevant to your study. If you are not sure if a list item applies to your research, read the appropriate section before selecting a response.

Materials & experimental systems

n/a	Involved in the study	
☐	☒	Antibodies
☒	☐	Eukaryotic cell lines
☒	☐	Palaeontology and archaeology
☒	☐	Animals and other organisms
☒	☐	Human research participants
☐	☒	Clinical data
☒	☐	Dual use research of concern

Methods

n/a	Involved in the study	
☒	☐	ChIP-seq
☒	☐	Flow cytometry
☒	☐	MRI-based neuroimaging

Antibodies

Antibodies used	All 99 whole tumour sections used in this study were dual stained for CA9 and FOXP3. CA9, rabbit ; Company: Sigma #HPA055207; stain: CYTO. FOXP3 mouse, Company: ABCAM #ab20034,Stain: NUC.
Validation	Formalin fixed paraffin embedded tissues were dewaxed and 5µm sections cut. Antigen retrieval was performed by steaming in 1X Citrate buffer (Sigma C9999). Dual staining was performed using the ImmPRESS Duet Double Staining Polymer kit (HRP Anti-Mouse

IgG/AP Anti-Rabbit IgG, Vector labs, MP-7724) as per manufacturer's instructions. Sections were stained for cytoplasmic CA9 expression (ImmPACT Vector Red, magenta; primary antibody: rabbit anti-CA9, Novusbio #NB100-417) and nuclear FOXP3 expression (ImmPACT DAB, brown; primary antibody: mouse anti-FOXP3, ABCAM #ab20034), followed by hematoxylin counterstain.

Clinical data

Policy information about clinical studies

All manuscripts should comply with the ICMJE guidelines for publication of clinical research and a completed CONSORT checklist must be included with all submissions.

Clinical trial registration	n/a- This is a non-interventional study and therefore not classified as a clinical trial.
Study protocol	TBCRC 038
Data collection	n/a
Outcomes	n/a
