# Supplementary material for: Spatial interplay of tissue hypoxia and T-cell regulation in ductal carcinoma in situ
Source: NPJ Breast Cancer. 2022 Sep 15;8:105. doi: 10.1038/s41523-022-00419-9 (PMC9477879; doi:10.1038/s41523-022-00419-9)
Supplement: Supplementary file 2 — Supplementary Information [file 41523_2022_419_MOESM2_ESM.docx]

**Spatial colocalization of CA9 and FOXP3 positive cells in pure DCIS and IDC/DCIS samples**


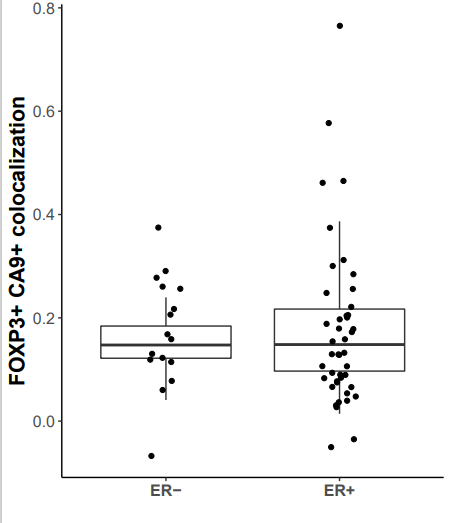
In our study, the average number of Foxp3+ cells (number of foxp3+ cells/total number of cells per sample) , ca9+ cells (number of ca9+ cells/total number of cells per sample) FOXP3+CA9+ colocalization does not vary significantly between ER+/- samples (p=0.11, p=0.39 and p = 0.82 respectively) (Figure 1).


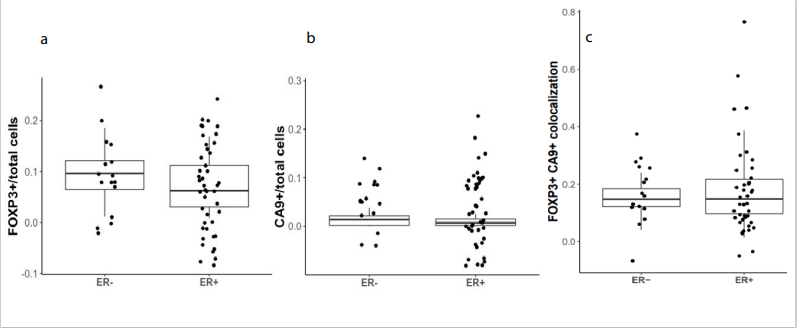


**Supplementary Figure 1.** Boxplots comparing the of a) Foxp3+ cells (number of foxp3+ cells/total number of cells per sample), (p=0.11), b) ca9+ cells (number of ca9+ cells/total number of cells per sample) (p=0.39), c) FOXP3+CA9+ colocalization (p = 0.82) between ER+/- samples.


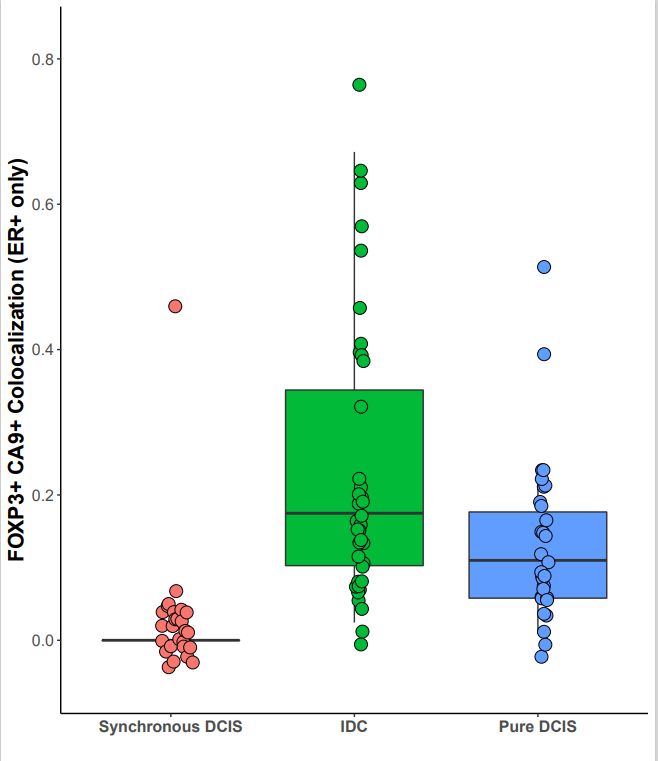
Our findings that FOXP3+CA9+ colocalization is significantly higher in IDC compared to pure-DCIS and synchronous DCIS holds true for ER+ samples. In other words, the increased colocalization between FOXP3+ and CA9+ cells is independent of ER status (Figure 2).

**Supplementary Figure 2.** Boxplots comparing the FOXP3+CA9+ colocalization for the ER+ samples in pure-DCIS, synchronous DCIS and IDC.
